# Supplementary material for: Single-cell RNA sequencing reveals cellular and molecular heterogeneity in fibrocartilaginous enthesis formation
Source: eLife. 2023 Sep 12;12:e85873. doi: 10.7554/eLife.85873 (PMC10513478; doi:10.7554/eLife.85873)
Supplement: Supplementary file 1. [file elife-85873-supp1.docx]

**Table Antibodies used in this study.**

| **Antibody (clone)** | **Vendor** | **Dilution** | **Usage** |
| --- | --- | --- | --- |
| Alexa Fluor® 700 anti-mouse TER-119 (TER-119) | Biolengend | 0.25ug/million cells | FACS |
| Alexa Fluor® 700 anti-mouse CD45 (I3/2.3) | Biolengend | 0.25ug/million cells | FACS |
| Alexa Fluor® 700 anti-mouse CD31 (390) | Biolengend | 0.25ug/million cells | FACS |
| DAPI | BD | 0.5ul/test | FACS |
| Anti-Rabbit Sox9 (ab185966) | Abcam | 1:200 | IF |
| Anti-Mouse Scx (sc518082) | Santa Cruz | 1:200 | IF |
| Anti-Rabbit Tnn (AB_2900654) | Thermo Fisher | 1:100 | IF |
| Anti-Rabbit Mfge8 (A12322) | Abclonal | 1:200 | IF |
| Anti-Rabbit Mfe2a/Mef2c (A2710) | Abclonal | 1:200 | IF |
| Anti-Rabbit Col2a1 (28459-1-AP) | Proteintech | 1:300 | IHC |
| Anti-Rabbit IgG (Alexa Fluor® 488) (ab150073) | Abcam | 1:400 | IF |
| Anti-Rabbit IgG (Alexa Fluor® 594) (ab150064) | Abcam | 1:400 | IF |
